# Biochemical and proteomic characterization of retrovirus Gag based microparticles carrying melanoma antigens

**DOI:** 10.1038/srep29425

**Published:** 2016-07-11

**Authors:** Reet Kurg, Olavi Reinsalu, Sergei Jagur, Kadri Õunap, Liisi Võsa, Sergo Kasvandik, Kärt Padari, Kiira Gildemann, Mart Ustav

**Affiliations:** 1 Institute of Technology, University of Tartu, Estonia; 2 Institute of Molecular and Cell Biology, University of Tartu, Estonia

## Abstract

Extracellular vesicles are membraneous particles released by a variety of cells into the extracellular microenvironment. Retroviruses utilize the cellular vesiculation pathway for virus budding/assembly and the retrovirus Gag protein induces the spontaneous formation of microvesicles or virus-like particles (VLPs) when expressed in the mammalian cells. In this study, five different melanoma antigens, MAGEA4, MAGEA10, MART1, TRP1 and MCAM, were incorporated into the VLPs and their localization within the particles was determined. Our data show that the MAGEA4 and MAGEA10 proteins as well as MCAM are expressed on the surface of VLPs. The compartmentalization of exogenously expressed cancer antigens within the VLPs did not depend on the localization of the protein within the cell. Comparison of the protein content of VLPs by LC-MS/MS-based label-free quantitative proteomics showed that VLPs carrying different cancer antigens are very similar to each other, but differ to some extent from VLPs without recombinant antigen. We suggest that retrovirus Gag based virus-like particles carrying recombinant antigens have a potential to be used in cancer immunotherapy.

Most cell types release extracellular vesicles which has a crucial role in both physiological and pathophysiological processes. Cell-derived membrane vesicles are endogenous carriers of proteins and nucleic acids that participate in transportation of these molecules between the cells and tissues. These membrane vesicles have been shown to be involved in intercellular communication^1^, coagulation^2^, tumorigenesis^3^ and in immune responses^4^, and have an emerging role in the biology of stem cells. Recently, extracellular vesicles have created an excitement in the field of drug delivery having the potential to be exploited for delivery of exogenous therapeutic cargo *in vivo*^5^.

Extracellular vesicle is a collective term used for any kind of secreted extracellular vesicles, including exosomes, microvesicles and apoptotic bodies. Microvesicles, sometimes also called microparticles, are plasma-membrane-derived particles with the size of 100–1000 nm in diameter that are released into the extracellular space by outward budding and fission of the plasma membrane. The release of cellular microvesicles appears to share similarities with the retrovirus budding^6^. Retroviruses assemble and bud through a series of distinct stages, in which virus Gag protein molecules are first targeted to membrane sites of assembly, and then coalescence into semispherical particles that distort the membrane, and finally are released from the cell when the membrane neck is pinched off behind the assembled particle^7^. The retrovirus Gag protein induces the spontaneous formation of microvesicles or virus-like particles (VLPs) when expressed in the mammalian cells, and these particles are released into the cell culture medium^8^,^9^,^10^. VLPs can be used to present foreign epitopes to the immune system. Gag-based VLPs are widely used as vaccines because of their ability to stimulate humoral and cellular immune responses in the absence of additional adjuvants^11^,^12^. Recent studies have shown that targeting of tumor antigens to the surface of extracellular vesicles can improve antigen immunogenicity and therapeutic efficacy^13^,^14^,^15^.

Cancer/testis antigens (CTA) represent a unique class of tumor antigens, which are expressed in a wide variety of malignant tumors, while their expression in normal tissues is mostly restricted to germ cells of the testis, fetal ovary and placenta. Their tumor-restricted expression pattern makes these proteins promising clinical reagents for anticancer immunotherapy^16^. Melanoma-associated antigens (MAGE) were initially discovered in melanoma patients as tumor expressing antigens that are recognized by cytotoxic T-lymphocytes^17^. MAGEA proteins are known to be highly expressed in a wide range of cancers^18^,^19^,^20^,^21^. They are highly immunogenic and therefore are considered as potential targets for cancer vaccines and/or immunotherapy^22^.

In the present study, we have generated VLPs using the budding properties of murine leukemia virus (MLV) Gag protein, and characterized them by biochemical and proteomic methods. Our specific aim was to find out whether it is possible to incorporate recombinant proteins into VLPs without fusing them to membrane proteins. Five different melanoma antigens, MAGEA4, MAGEA10, MART1, TRP1 and MCAM, were co-expressed with the MLV Gag protein in mouse fibroblast cells, and their incorporation into VLPs and localization within the particles were determined. MAGEA4 and MAGEA10 are cancer-testis antigens expressed in a wide range of cancers with the limited expression in normal tissues^23^. MART1 (MELAN-A) and TRP1 or tyrosinase-related protein (gp75) are melanoma antigens expressed both in melanocytes and melanomas^24^, and MCAM (MUC18, CD146) is a melanoma cell adhesion molecule that is abnormally expressed in a variety of tumors and is closely associated with metastasis^25^. Our data show that the MAGEA4 and MAGEA10 proteins as well as MCAM are expressed on the surface of VLPs. The compartmentalization of exogenously expressed cancer antigens within the VLPs did not depend on the localization of the protein within the cell. Comparison of the protein content of VLPs showed that VLPs carrying different cancer antigens are very similar to each other, but differ to some extent from VLPs without recombinant antigen. We suggest that MLV Gag induced particles carrying recombinant antigens have a potential to be used in cancer immunotherapy.

## Results

### Generation and purification of virus-like particles carrying melanoma antigens

**Figure 1:**
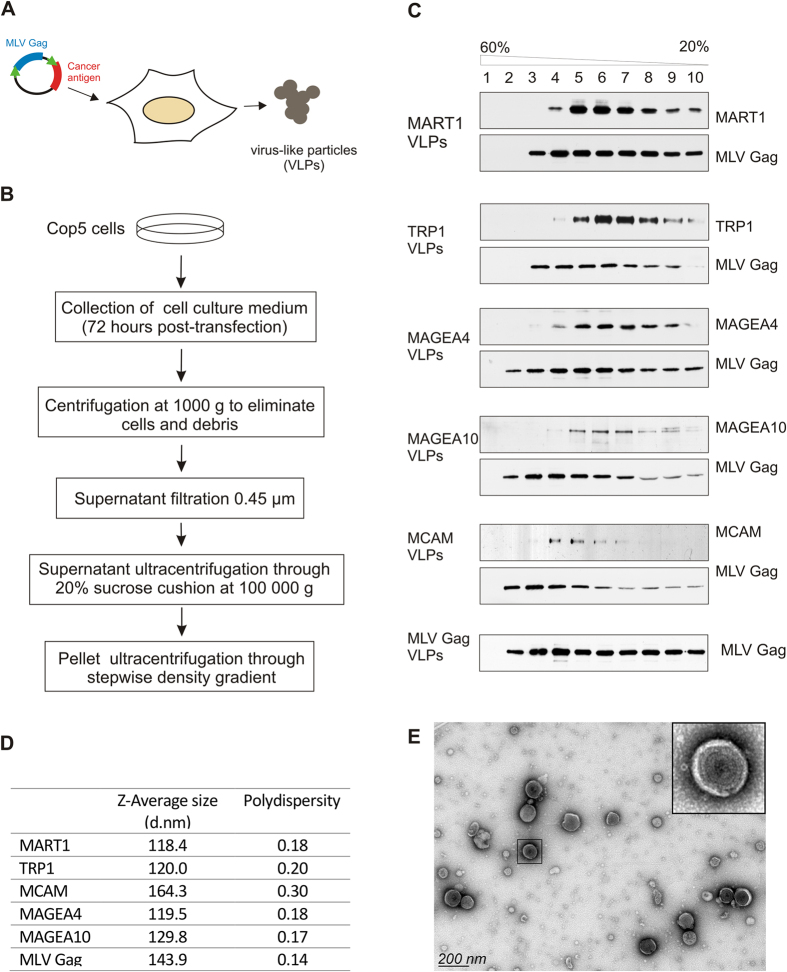
Generation and purification of VLPs. (**A**) General workflow of generation of MLV Gag based VLPs. (**B**) Schematic representation of the purification protocol. (**C**) Western blot analysis of VLPs. Particles obtained with ultracentrifugation through 20% sucrose cushion were further centrifuged through stepwise sucrose density gradient (20%, 35%, 45%, 60%) at 120 000 g and 4 °C for 1.5 h in a Beckman SW55 rotor and divided into 10 fractions. The presence of VLPs in each fraction was analyzed by specific antibodies against melanoma antigens and the MLV Gag protein. (**D**) Physical characterization of VLPs ultracentrifuged through 20% sucrose cushion as assessed by DLS. The mean value of 4 × 10 measurements performed at 22 °C is shown. (**E**) Transmission electron micrograph of negatively stained MAGEA4 VLPs purified through density gradient centrifugation. Enlarged image of particle is shown on the upper-right corner of the picture.

In order to generate MLV Gag based VLPs, we transfected the mouse fibroblast COP5 cells with DNA plasmids encoding for codon-optimized MLV Gag protein with or without coding sequences for five different melanoma antigens (Fig. 1A). Throughout the study, the MLV Gag refers to VLPs obtained by expression of MLV Gag protein without any recombinant cancer antigen, and MART1, TRP1, MAGEA4, MAGEA10 and MCAM mark the VLPs carrying respective cancer antigens. The VLPs were purified from the cell culture media 72 hours after electroporation according to the scheme shown in Fig. 1B. First, the cell culture media was depleted of intact cells and cell debris by low-speed centrifugation and filtration through a 0.45 μm membrane. Next the culture media was ultracentrifuged through 20% sucrose cushion at 100 000 g resulting in sedimentation of VLPs to the bottom of the tube. We also performed the second ultracentrifugation step through stepwise sucrose density gradient in order to get rid of membraneous particles or membrane fragments and protein aggregates which may have co-sedimented with VLPs through 20% sucrose cushion. This step also allowed us to compare the centrifugation profile of VLPs carrying different melanoma antigens. As shown in Fig. 1C, the MLV Gag protein was detected in several fractions starting from 2 with a peak in fractions 3–6. Similar distribution of the Gag protein was detected in case of all VLPs. The distribution of melanoma antigens was mostly similar, however some differences were observed. The MCAM VLPs were detected in fractions 4–6 similar to MLV Gag VLPs, while MART1, TRP1, MAGEA4 and MAGEA10 VLPs peaked in fractions 5–7 (Fig. 1C). In spite of these small differences, the Gag protein and melanoma antigens were detected in same fractions suggesting that Gag-induced VLPs carry recombinant antigens.

Next we performed a dynamic light scattering (DLS) analysis to characterize the size distribution of Gag-based VLPs. The hydrodynamic diameters (Z-average size) and polydispersity indexes (Pdi) of VLPs purified by ultracentrifugation through 20% sucrose cushion are shown in Fig. 1D and the bell-shaped size distribution profile in Fig. S1. MART1, TRP1, MAGEA4 and MAGEA10 VLPs had very similar particle sizes and Pdi-s, ranging from 118–130 d.nm with Pdi-s from 0.17 to 0.20. MLV Gag VLPs formed a peak at 144 d.nm (Pdi 0.14) and MCAM VLPs at 164 d.nm (Pdi 0.3). These data refer to homogenous preparations of VLPs. We also compared the dynamic diameter and Pdi of VLPs purified through stepwise density sucrose gradient with those VLPs which were purified through 20% sucrose cushion only and found them to be very similar with each other (Fig. S1).

To characterize the VLPs in more detail, we pooled fractions 4–7 of each sample and used them for morphological analysis by transmission electron microscopy (TEM). Analysis of the negatively stained VLP samples revealed the presence of membrane particles with diameter ranging from 100 to 140 nm (Fig. 1E). VLPs formed by over-expression of MLV Gag protein appeared as spherical structures with regular shape and size (Fig. 1E; enlarged image). In all probes, we also detected some VLPs with interrupted or loosely packed membranes, which may refer to immature VLPs or resulted of their fragile nature. The VLPs carrying different cancer antigens looked very similar to each other and to particles induced by MLV Gag protein alone as revealed by visual observation in electron microscope. Analysis of the samples also showed the presence of smaller membrane particles with diameters from 40 to 80 nm, but no larger particles (larger than 150 nm) were observed.

### MAGEA proteins are exposed on the surface of VLPs

**Figure 2:**
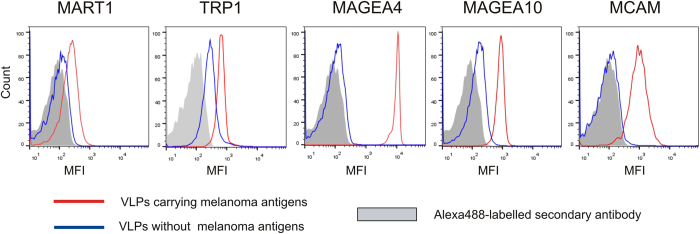
Surface analysis of melanoma antigens incorporated into VLPs. FACS analysis was performed with VLPs bound to aldehyde/sulfate latex beads as described in Materials and Methods section. The red line corresponds to VLPs carrying recombinant melanoma antigens, the blue line to VLPs without cancer antigen, and grey area shows the signal obtained with secondary Alexa488-labelled antibody. One representative experiment out of the three performed is shown. MFI = Mean fluorescence intensity.

In order to study the surface expression of melanoma antigens, we performed immunostaining coupled to flow cytometry analysis of VLPs bound to aldehyde/sulfate latex beads. The results of a representative experiment out of the three performed with similar results are shown in Fig. 2. MAGEA4, MAGEA10 and MCAM antibodies all bound very efficiently to respective VLPs (red line) and gave much lower signal with VLPs formed by expression of MLV Gag protein alone (blue line). The highest fluorescence signal was observed for the MAGEA4 protein, followed by MAGEA10 and MCAM proteins (Fig. 2). Antibodies specific to MART1 and TRP1 also recognized the VLPs carrying MART1 or TRP1 proteins, respectively; however, to much lesser extent. It is possible that these proteins are packed within the VLPs and only a portion of them is exposed on the surface of VLPs. However, we cannot rule out the possibility that MART1 and TRP1 proteins are expressed on the surface of particles, but the antibodies used in this study poorly recognize their surface epitopes. The TRP1-specific antibody also gave some signal with MLV Gag VLPs suggesting that this antibody cross-reacts with particle membrane. Similar results were obtained with VLPs purified through 20% sucrose cushion as well as with VLPs ultracentrifuged through sucrose cushion and density gradient.

### Localization of melanoma antigens within the cells

**Figure 3:**
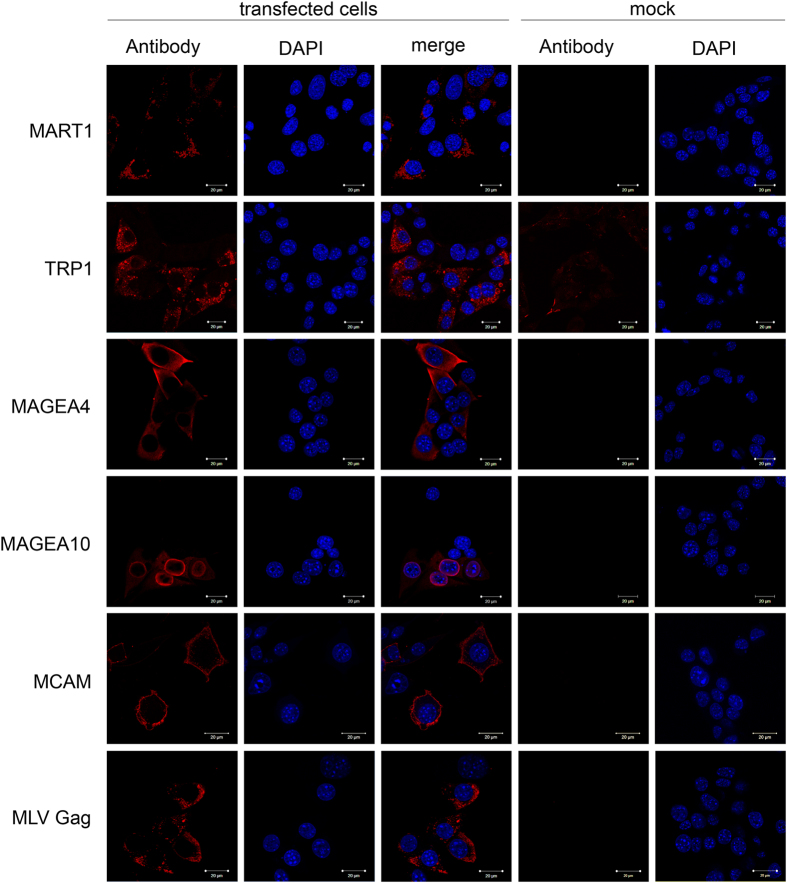
Subcellular localization of melanoma antigens used in this study. The COP5 cells transfected with plasmids encoding for the MLV Gag protein and respective cancer antigen were grown on cover slips, fixed and incubated with specific antibodies and Alexa568-conjugated secondary antibody as described in Methods section. Untransfected cells (mock control) incubated with specific antibodies are shown. DAPI was used to stain nuclei of the cells.

In order to analyze whether the surface expression of MAGEA proteins in VLPs is dependent on their localization within the cells, we first performed indirect immunofluorescence analysis in mouse fibroblast cells. According to this assay, the MAGEA4 protein is localized to the cytoplasm of the cell with no obvious preference to any specific compartment (Fig. 3; MAGEA4 panel). In some cells, MAGEA4 was detected as a diffuse signal near to the plasma membrane. MAGEA10 was observed in the nucleus of the cell consistent with previously published results^26^. The MAGEA10 protein accumulated to the nuclear membrane and was also detected in the cytoplasm in mouse fibroblast cells used in this study. MCAM is a cell surface glycoprotein which plays role in cell adhesion. Accordingly, the MCAM protein expression was detected in the plasma membrane (Fig. 3). MART1 and TRP1 are both involved in melanin biosynthesis and are localized in endoplasmic reticulum membrane and Golgi area^27^,^28^ and our indirect immunofluorescence analysis confirmed the previous results (Fig. 3). MART1 and TRP1 localization was also detected in the membrane of small vesicles in the cytoplasm of the cells, but was not observed in the plasma membrane (Fig. 3). TRP1 antibody showed some cross-reactivity with membrane as seen in the panel of mock control. The MLV Gag protein was localized in granular structures throughout the whole cytoplasm. Co-localization of the MLV Gag protein with any of the melanoma antigens used in this study was not detected.

**Figure 4:**
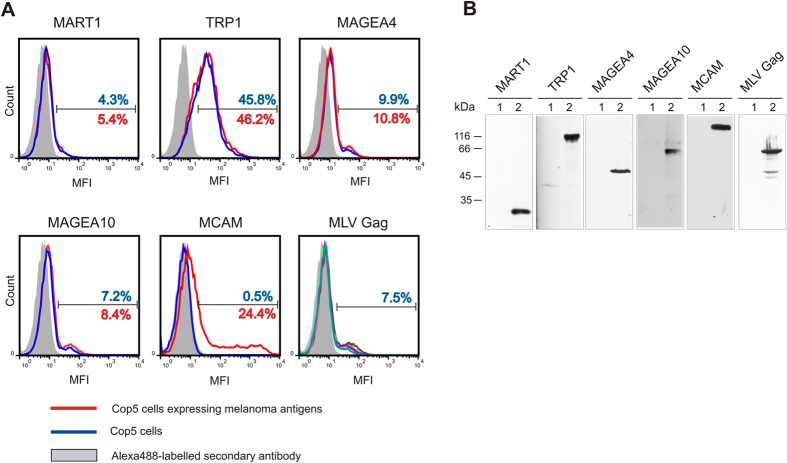
Cell surface expression of melanoma antigens transiently expressed in COP5 cells. (**A**) FACS analysis was performed with immunostained living cells transfected with plasmids encoding for cancer melanoma antigens MART1, TRP1, MCAM, MAGEA4 and MAGEA10, and MLV Gag. The red line corresponds to cells expressing recombinant melanoma antigens, the blue line to cells without cancer antigen, and grey area shows the signal obtained with secondary Alexa488-labelled antibody. The percentage of gated cells is shown (blue for cells without cancer antigen and red for cells expressing recombinant melanoma antigens) on the image. The image of MLV Gag contains the data of five transfections obtained with electroporation with five different melanoma antigens. In all cases, one representative experiment out of the two performed is shown. MFI = Mean fluorescence intensity. (**B**) Western blot analysis of COP5 cells expressing melanoma antigens. Lane 1, mock control; lane 2, transfected cells.

To analyze the expression of melanoma antigens on the cell surface in transient over-expression experiments, we have also performed flow cytometry analysis with living cells. For this, we transfected the COP5 cells with plasmids encoding for melanoma antigens and MLV Gag protein, and stained the cells with respective antibodies. As shown in Fig. 4, none of the melanoma antigens, except for the MCAM protein, were exposed on the cell surface of fibroblasts expressing the melanoma antigens and MLV Gag protein (Fig. 4A). In the case of MCAM, 25% of cells were positively stained with the specific antibody (red line); this is consistent with the transfection efficiency of fibroblasts and suggests that the MCAM protein is exposed on the external side of plasma membrane. The MLV Gag protein is known to bind to the internal side of plasma membrane and was not detected on the surface of transfected cells. The antibodies generated against the TRP1 protein bound to the cell membrane of TRP1-MLV Gag as well as MLV Gag transfected cells, suggesting that they cross-react with some cell surface proteins. All the melanoma antigens were expressed with expected sizes in COP5 cells (Fig. 4B).

Taken together, our data suggest that the MAGEA4, MAGEA10 and MCAM proteins are expressed on the surface of Gag-induced VLPs. This localization does not depend or correlate with the localization of the respective proteins within the cells as all these three proteins differ in their subcellular localization; MAGEA4 is expressed in the cytoplasm, MAGEA10 in the nucleus and MCAM on the outer membrane of the cell.

### Proteomic analysis of VLPs

**Figure 5:**
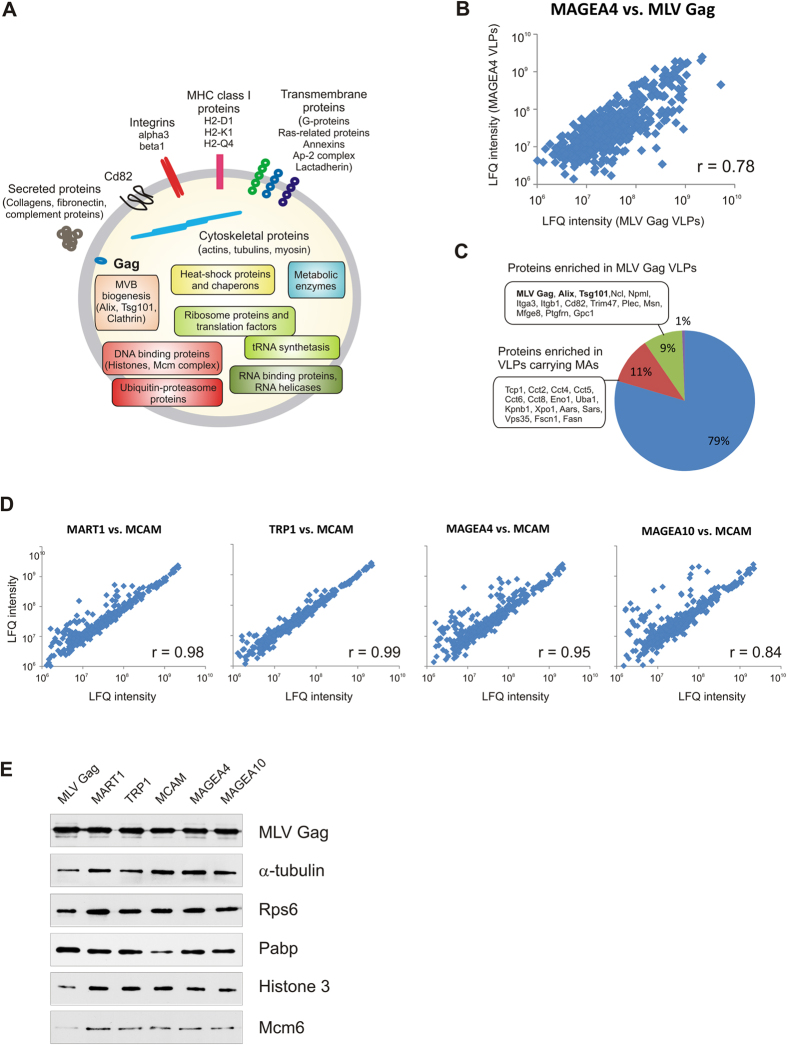
Proteomic analysis of VLPs. (**A**) The composition of MLV Gag induced VLPs. (**B**) Correlation of protein abundances (LFQ intensity) between VLPs carrying MAGEA4 antigen with VLPs without recombinant antigen (MLV Gag). Pearson correlation coefficient is shown on the blot. (**C**) Differently expressed proteins in MLV Gag VLPs and VLPs carrying melanoma antigens. (**D**) Comparison of LFQ intensities of VLPs carrying different cancer antigens. (**E**) Western blot analysis of VLPs using antibodies against MLV Gag and cellular proteins α-tubulin, Rps6, Pabp, histone H3 and Mcm6. The equal amount of total protein (100 ng for MLV Gag and 2 μg for others) was loaded on each lane.

In order to characterize the protein content of VLPs, LC-MS/MS-based label-free quantitative proteomics analysis was performed. All five melanoma antigens, the MART1, TRP1, MCAM, MAGEA4 and MAGEA10 proteins were detected in respective VLP’s confirming once again the effective incorporation of recombinant proteins into VLPs induced by overexpression of the MLV Gag protein. The Gag protein itself was one of the most abundant proteins of VLPs by proteome analysis (by Coomassie staining of the gels). We also identified more than five hundred unique cellular proteins with the criteria of at least two unique peptides per sample. All these cellular proteins were detected in all VLP samples. Identified proteins were divided into groups according to their biological function using the UniProt (www.uniprot.org) database (Fig. 5A; Table S1). In addition to the Gag protein, we identified several Gag interacting proteins which are essential for viral budding, such as Tsg101, Alix, Nedd4 and clathrin adaptor-protein complex AP2 and AP1 subunits^29^,^30^, as components of VLPs. It has been shown that retroviruses employ clathrin to facilitate the accurate morphogenesis of infectious particles^31^ and clathrin was one of the abundant cellular proteins in our proteomic screen. Most of the host proteins identified by LC-MS/MS correspond to intracellular proteins presumably located inside the VLPs. These include proteins involved in various cytoplasmic systems such as cytoskeleton (actin, moesin), microtubules (tubulin), signaling, metabolism, chaperons, ubiquitin-proteasome system, protein and vesicle trafficking. We identified numerous ribosome proteins, translation factors, RNA helicases and Hnrnp proteins as well as tRNA synthetases in our screen. tRNA synthetases were recently shown to be the binding partners of HIV Gag protein^32^. Additionally, the VLP preparations contained several nuclear proteins including histones and six subunits of the Mcm complex.

To assess the quantitative protein variability of VLPs, we have used the MaxQuant-based label-free analysis (LFQ intensities). Comparison of the relative abundances of proteins in VLPs without antigen (MLV Gag) with those of carrying different cancer antigens showed reasonable correlation with Pearson’s correlation coefficients of r = 0.67 (MCAM vs. MLV Gag), 0.70 (TRP1 vs. MLV Gag), 0.74 (MAGEA10 vs. MLV Gag), 0.75 (MART1 vs. MLV Gag) and 0.78 (MAGEA4 vs. MLV Gag) (Fig. 5B). Most of the cellular proteins were equally distributed in all VLPs. However, approximately 20% of proteins were differently regulated in VLPs carrying melanoma antigens compared to MLV Gag VLPs when the cut-off value was set as 3-fold difference in LFQ values (Fig. 5C). Comparison of LFQ intensities of VLPs carrying cancer antigens showed high correlation coefficients (Fig. 5D) indicating a good overlap of proteins between VLPs with different recombinant melanoma antigens. The biggest differences in protein abundance were observed between MAGEA10 and MCAM VLPs, but no specific protein groups were identified. These data show that the VLPs carrying cancer antigens are more similar to each other than to MLV Gag VLPs. We also performed western blot analysis with independently purified VLPs using antibodies against some cellular proteins including cytoskeleton proteins α-tubulin, ribosomal protein Rps6 and polyA binding protein Pabp, and nuclear proteins histone H3 and Mcm6 (Fig. 5E). The equal amount of total protein was loaded on each lane to compare the relative abundance of these proteins between different VLPs. As shown in Fig. 5E, the MLV Gag protein was detected on the same level in all VLPs, while histone H3, Mcm6, Rps6 and α-tubulin were detected on lower level in VLPs without recombinant melanoma antigens (MLV Gag; first lane). This suggests that the relative abundance of MLV Gag protein is higher in MLV Gag VLPs than in other VLPs and is consistent with the MS data. At the same time, we did not detect any major differences in the relative abundance of cellular proteins between VLPs carrying different melanoma antigens.

## Discussion

In this study, we have employed the robust particle budding properties of the MLV Gag protein for production of VLPs. We show that the cancer/testis antigens MAGEA4 and MAGEA10 are incorporated on the surface of Gag-based particles when transiently expressed together with the MLV Gag protein. This makes them attractive vaccine candidates for cancer immunotherapy. Antitumor vaccination is a promising tool to induce systematic immunity against malignant cells, and MAGEA proteins with their unique expression pattern are good candidates for it^33^,^34^.

VLPs can be used to present foreign epitopes to the immune system. This is usually achieved by genetic fusion or chemical conjugation between target antigens and structural viral proteins that can self-assemble into VLPs^35^,^36^,^37^. Several studies have shown that the immunogenicity of antigens can be improved by targeting their localization to extracellular vesicles like exosomes, which are small, 50 to 100 nm vesicles secreted by most cell types. In the technology platform called exosome display, the antigen is fused to the C1C2 domain of lactadherin, which is homologous to the C-terminal domain of blood coagulation factor V and factor VIII, and binds to phosphatidylserine exposed at the surface of apoptotic cells or dendritic cell derived exosomes, increasing vaccine potency^15^,^36^,^38^,^39^. Retrovirus Gag based VLPs have been successfully used in HCV and HIV vaccine development. RetroVLPs pseudotyped with either HCV-E1E2 or –E1 proteins induced high-titer antibodies, including neutralizing antibodies, in both mouse and macaque^40^,^41^. VLPs derived from enveloped viruses offer the unique opportunity to integrate target antigens displayed onto the particles by their integration in the envelope that is cell membrane. This is usually achieved by rationale vaccine design by fusion of antigen of interest with the transmembrane domain of virus glycoproteins or C1C2 domain of lactadherin^12^,^36^,^41^. In our work, we show that the MAGE4, MAGEA10 and MCAM proteins are localized to the surface of VLPs without any additional genetic or chemical manipulations. MCAM is a glycosylated cell adhesion molecule expressing on the surface of the outer membrane of the cell, and its localization on the surface of VLPs was predictable. On the other hand, we expected that the MART1 and TRP1 proteins, localized to the inner membranes (endoplasmatic reticulum) of the cell, are also exposed on the surface of VLPs, but were not able to show this. At the same time, the presentation of MAGEA proteins on the surface of VLPs was unpredictable and surprising. The mechanism of surface expression of MAGEA proteins is not known and is under further investigation.

The MAGEA antigens are of particular interest for cancer immunotherapy because they are strictly tumor specific and are shared by many kind of tumors^22^. MAGEA4 and MAGEA10 belong to MAGEA family of proteins comprised of 12 members called MAGEA1-MAGEA12^23^. The MAGEA proteins share a common MAGE homology domain (MHD), which can interact with variable domains in RING finger E3 ubiquitin ligases enhancing the ubiquitin ligase activity of RING domain proteins^42^,^43^. Several MAGEA proteins control the function of p53 tumor suppressor by inhibiting p53 transactivation function, either by recruiting histone deacetylases^44^ or by interacting with the DNA binding surface of p53^45^. In addition, some MAGEA proteins are involved in the regulation of apoptosis and cell cycle. The carboxy-terminal 107 amino acids of MAGEA4, but not the whole protein, were found to induce apoptosis through p53-dependent and independent pathways^46^. In another work, MAGEA4 overexpression increased the apoptotic index in HEK293 cells^19^. However, none of these activities does explain the expression and presentation of MAGEA4 protein on the surface of VLPs.

We have carried out the proteomic characterization of MLV Gag based VLPs and identified more than five hundred proteins as components of VLPs. Retroviruses are known to incorporate cellular proteins on the surface of and inside the virion^47^,^48^. Host proteins can be incorporated into viral particles either randomly, because they are present at the site of virus budding, or specifically, because they interact with certain virus constituents^49^. Active incorporation of proteins into Gag based VLPs requires interactions between the viral Gag protein and/or recombinant proteins incorporated into the particles. We identified several proteins essential for viral budding, such as Tsg101, Alix, Nedd4 and clathrin adaptor-protein complex AP2 and AP1 subunits^29^,^30^ and the Gag protein interactors such as Ppia (Cyclophilin A)^50^, and the tRNA synthetase complex^32^ in our VLP samples. Recent studies have also shown that MLV particles acquire actin and the actin-binding protein moesin^51^, and ubiquitin^52^. On the other hand, a lot of the proteins of VLPs are cytoplasmic proteins that are commonly identified by different studies of exosomes and other extracellular vesicles. These include cytoskeletal proteins (actins, tubulins, moesin etc.), metabolic enzymes (GAPDH, enolase, PKM2), ribosomal and heat shock proteins. We have also identified several cell surface and adhesion proteins, namely, integrins α3 and β1, lactadherin Mfge8, annexins, MHC I proteins etc. Identification of membrane proteins is particularly attractive since they could serve as targets for purification of vesicles or for the insertion of tags for purification, labeling, or targeting purposes. In our study, we could not identify tetraspanin molecules CD9, CD63 and CD81 used for affinity purification and detection of exosomes. At the same time, we detected a large amount of proteasome proteins, the Mcm complex and a lot of histones in our samples, which raises the question whether they are incorporated into Gag-induced VLPs or are simply co-purifying with them. We cannot rule out the possibility that small apoptotic bodies or their fragments or membrane enclosed vesicles containing proteasomal subunits^53^ are co-sedimenting with Gag-induced VLPs. Limitations of the analysis of the proteome of extracellular vesicles are largely due to difficulties in getting highly purified samples. Cells release a mixture of extracellular vesicles (microparticles, exosomes and apoptotic bodies) that are to some extent similar in density and composition. Additionally, exosomes and retroviruses may use the same endosomal pathway during the budding process and incorporate similar cellular proteins. Furthermore, analysis of profiles of host proteins packed into the HIV virions have shown that the content of virions is dependent on the type of virus producing cell. For example, only 42 proteins of 202 were detected in all cases in the core of HIV virions using three different cell types^54^. In addition, analysis of incorporation of tetraspanins have shown the dynamic nature of host-derived protein incorporation into MLV VLPs^55^. Different purification protocols have been validated and compared^56^,^57^, but still it is sometimes difficult to say, which proteins are incorporated into microvesicles and which have co-purified with them.

New cancer therapies are urgently needed, since available treatment options today have sometimes negative side effects, and cure only about half of the patients suffering from invasive cancer. One, relatively new, option is to vaccinate against cancer, by introducing an antigen that is present on the tumor cells into the patient to stimulate specific immunity against the tumor. One possibility among others is to use virus-like particles exposing cancer antigens on their surface for this purpose.

## Methods

### Plasmids and cell culture

The coding sequences of MART1 (also known as MLANA; NM_005511), TRP1 (TYRP1; NM_000550), MAGEA4 (NM_002362), MAGEA10 (NM_021048) and MCAM (NM_006500) were amplified by PCR using specific primers and cloned into the pQMCF (Icosagen AS, Tartu, Estonia) expression vector under the control of hEF1a promoter. All these plasmids contain the second expression cassette enabling the expression of the MLVGag protein from the CMV promoter (Icosagen AS). The COP5-EBNA cells obtained from Icosagen AS (Tartu, Estonia) were grown in Iscove’s Modified Dulbecco’s Medium (IMDM) supplemented with 10% fetal calf serum (FCS), 100 U/ml penicillin and 100 μg/ml streptomycin. For DNA electroporation, 250 μl of cell suspension in IMDM was mixed with 2 μg of expression plasmid and 50 μg of salmon sperm carrier DNA, and transfected by electroporation in 4-mm cuvettes (Thermo Fisher Scientific) using Bio-Rad GenePulser Xcell (settings 230 V, 975 μF).

### Proteins and antibodies

Generation of antibodies against MART1, TRP1, MAGEA4 and MAGEA10 proteins was carried out by Icosagen AS. In this work, the affinity purified antibodies from rabbit serums were used. For generation of antibodies, recombinant N-His-tagged MAGEA4 and MAGEA10 proteins were expressed in *Escherichia coli* cells BL-CodonPlus™RP (Invitrogen) and purified with Ni-Sepharose™6 Fast Flow beads (GE Healthcare) under native conditions. TRP1 was produced in mammalian CHO cells as the truncated protein (aa 1–477) fused to the C-terminal His-tag by Icosagen AS. TRP1(1–477)-His protein was purified from cell culture media using Ni-Sepharose™6 Fast Flow beads (GE Healthcare). The cytoplasmic domain of MART1 (aa 48–118) fused to the mouse IgG2a Fc domain was also produced in CHO cells by Icosagen AS and purified with Protein A Sepharose CL-4B beads (GE Healthcare). In all cases, after purification the buffer was exchanged to PBS with Amicon^®^Ultra centrifugal filters (Millipore) and the concentration of proteins was determined by Bradford assay using BSA as a standard.

### Generation and purification of VLPs

The cell culture medium of COP5-EBNA cells electroporated with pQMCF plasmid encoding for melanoma antigen and MLV Gag protein was collected three days after transfection and purified from cell debris by centrifugation at 1000 g for 10 minutes at room temperature and filtered through 0.45 μm syringe filters by gentle pressure. Then, filtered samples were centrifuged at 100 000 g and 4 °C for 3 h through 5 ml of 20% sucrose cushion in PBS in a Beckman SW28 rotor. The pellets were resuspended in 300 μl of TN buffer (0.05 M Tris-HCl; pH 7.5, 0.1 M NaCl) overnight at 4 °C. For second ultracentrifugation, 250 μl of each VLP sample was layered on the top of the stepwise gradient consisting of 1 ml of 20%, 35%, 45% and 60% sucrose in PBS and centrifuged at 120 000 g and 4 °C for 1.5 h in a Beckman SW55 rotor. Gradient was divided into 10 fractions and analyzed by western blotting. For further analysis, positive fractions were pooled and concentrated by Amicon^®^Ultra centrifugal filters (0.5 ml, cut-off 100 KDa; Millipore) according to manufacturer’s manual. The concentration of total proteins was determined by Bradford assay using BSA as a standard.

### Transmission electron microscopy (TEM)

The VLPs carrying different melanoma antigens were visualized using negative staining transmission electron microscopy (TEM). The copper grids covered with formvar film and carbon layer were applied onto the drops of sample solution for 5 min. The excess solution was removed with filter paper; grids were briefly washed with Milli-Q water and transferred onto the drops of 2% aqueous uranyl acetate solution for 30 sec. After removing the excess stain, samples were allowed to air dry. TEM analysis was performed using FEI Tecnai G2 Spirit BioTWIN transmission electron microscope (FEI, The Netherlands) run at 120 kV. The images were recorded ith Orius SC1000 CCD camera (Gatan Inc, USA) and processed with Adobe Photoshop CS4.

### Dynamic Light Scattering

DLS measurements were performed with Zetasizer Nano (Malvern Instruments, UK). 4 × 10 measurements were performed with following settings (refractive Index = 1.330, viscosity = 0.955, temperature = 22 °C) in 70 μl with VLPs having total protein concentration between 0.5–0.7 mg/ml. The diameter of particles was calculated by Zetasizer software using sphere approximation.

### Flow Cytometry

For living cell analysis, COP5-EBNA cells transfected with expression plasmids were collected 24 h post-transfection and suspended in 1 ml of ice-cold 10% FCS in PBS. Then 100 μl of blocking solution (5% BSA in PBS) was added and cells were incubated for 30 min with end-over-end rotation. Cells were centrifuged at 400 g for 5 min and resuspended in 3% BSA/PBS containing affinity purified antibodies. The final concentrations of antibodies were following: 1 μg/ml for anti-MAGEA4 and 2 μg/ml for anti-MAGEA10, anti-MART1, anti-TRP1 (this study), and anti-MCAM (sc-18837 Santa Cruz). After 1 h incubation with rotation in the dark, and three times of washing with 3% BSA/PBS, the cells were incubated with Alexa488-conjugated goat anti-rabbit antibody (Invitrogen) at dilution 1:1000 in the dark at 4 °C. Cells were washed, resuspended in 500 μl of 10% FCS/PBS and analysed by flow cytometry (LSRII; BD Biosciences) using the FACSDiva software (BD Biosciences). Analysis was performed with FlowJo VX (Tree Star).

For analysis of VLPs, 30 μg of VLPs (according to total protein) were incubated with 10 μl of 4 μm diameter aldehyde/sulfate latex beads (Invitrogen) for 15 min at room temperature. Then PBS was added to the final volume of 1 ml and incubation was continued overnight at 4 °C in the dark using end-over-end rotation. The beads were blocked with 100 mM glycine in PBS at 4 °C for 30 min and then washed three times with 2% BSA in PBS. Incubation with antibodies was carried out in 2% BSA in PBS for 1 hour in the dark using end-over-end rotation. After three times of washing, the secondary antibody conjugated with Alexa488 was added and incubated again for 1 hour in the dark using end-over-end rotation. Beads were washed, resuspended in 500 μl of 2% BSA in PBS and analysed by flow cytometry as described above.

### Immunofluorescence and immunoblotting

For immunofluorescence analysis, COP5-EBNA cells transfected with expression plasmids were grown on glass coverslips. Forty-eight hours post-transfection the cells were washed with PBS, fixed in 4% paraformaldehyde in PBS for 10 min at room temperature and permeabilized with 0.5% Triton X-100 in PBS for 5 minutes at room temperature. Cells were blocked in PBS containing 5% bovine serum albumin and incubated with affinity-purified rabbit polyclonal antibodies against MART1, TRP1, MAGEA4, MAGEA10 (final concentration of 5 μg/ml), MLVGag (2 μg/ml; Icosagen AS) and MCAM (2 μg/ml; sc-18837 Santa Cruz) in 1% BSA in PBS for 1 hour, and with Alexa-568 conjugated secondary antibody (dilution 1:1000) (Invitrogen), washed in PBS/0.1% Tween20 and placed under coverslips with SlowFade^®^ Gold antifade reagent with DAPI (Invitrogen). Analysis was performed using confocal laser scanning microscope LSM710 (Zeiss). Images were obtained with 63× lens and analysed by ZEN2011 software.

For immunoblot analysis, cells were lysed and proteins were separated by SDS-polyacrylamide gel electrophoresis and transferred by a semidry blotting method to a polyvinylidene difluoride (PVDF) membrane (Millipore Corp.). Membranes were incubated with affinity-purified rabbit polyclonal antibodies against MART1 (final concentration of 0.5 μg/ml), TRP1 (0.4 μg/ml), MAGEA4 (0.1 μg/ml), MAGEA10 (0.6 μg/ml), or with commercially obtained MLVGag (0.3 μg/ml; Icosagen AS), MCAM (0.1 μg/ml; sc-18837 Santa Cruz), α-tubulin (1/10000; T5168, Sigma), Rps6 (1/250; sc-74459, Santa Cruz), Pabp (1/500; ab21060, Abcam), Histone H3 (1/500; 06–599, Millipore) and Mcm6 (1/500; sc-9843, Santa Cruz), and secondary goat anti-rabbit or anti-mouse antibodies, respectively, conjugated with HRP. Detection was performed using an ECL detection kit (GE Healthcare) following the manufacturer’s manual.

### Proteomic analysis

VLPs were precipitated with 10% (v/v) trichloroacetic acid and suspended in 7 M urea, 2 M thiourea and 100 mM ammonium bicarbonate (ABC) digestion buffer. Proteins were reduced with 2.5 mM dithiothreitol and alkylated with 5 mM iodoacetamide, followed by 4 h predigestion with 1:50 (enzyme to protein) Lys-C (Wako Pure Chemical Industries) at room temperature. The sample was diluted five times with 100 mM ABC and further digested with 1:50 trypsin (Sigma Aldrich) overnight at room temperature. Peptides were cleaned on in-house made C18 (3 M Empore) StageTips and reconstituted in 0.5% trifluoroacetic acid.

Peptides were injected to an Agilent 1200 series nano-LC with an in-house packed (3 μm ReproSil-Pur C18AQ particles) 15 cm × 75 μm ID emitter-column (New Objective). Separation was carried out with an 8–40% gradient of buffer B at 250 nl/min for 2 h (buffer A: 0.5% AcOH, buffer B: 80% ACN, 0.5% AcOH) and the peptides were detected (spray voltage 2.0–2.2 kV) with an LTQ Orbitrap XL (Thermo Fisher Scientific) mass-spectrometer (MS). Each MS scan was followed by MS/MS analysis of the 5 most intense peaks. Dynamic exclusion was set to 60 s and only charge states over +1 were analysed. Mass-spectrometric raw data were analyzed with MaxQuant 1.4.0.8^57^,^58^. Two missed cleavages were allowed. Carbamidomethylation was set as a fixed modification and methionine oxidation, N-terminal acetylation as variable modifications. Data were searched against UniProtKB (www.uniprot.org) mouse (canonical and isoform) sequences. First and main search MS mass tolerances were ≤20 and ≤4.5 ppm, respectively. MS/MS tolerance was ≤20 ppm. Criteria for identification were specified as following: 1 peptide, minimum length of 7 residues and false discovery rate of <1% using a target decoy approach. Match between runs was enabled. All other parameters were default.

## Additional Information

**How to cite this article**: Kurg, R. *et al*. Biochemical and proteomic characterization of retrovirus Gag based microparticles carrying melanoma antigens. *Sci. Rep.* **6**, 29425; doi: 10.1038/srep29425 (2016).

## Supplementary Material

Supplementary Information

Supplementary Dataset 1
