# Supplementary material for: Biochemical and proteomic characterization of retrovirus Gag based microparticles carrying melanoma antigens
Source: Sci Rep. 2016 Jul 11;6:29425. doi: 10.1038/srep29425 (PMC4941533; doi:10.1038/srep29425)
Supplement: Supplementary Information [file srep29425-s1.pdf]

Supplementary Information

Biochemical and proteomic characterization of retrovirus Gag based microparticles carrying melanoma antigens

Reet Kurg, Olavi Reinsalu, Sergei Jagur, Kadri Õunap, Liisi Võsa, Sergo Kasvandik,
Kärt Padari, Kiira Gildemann and Mart Ustav

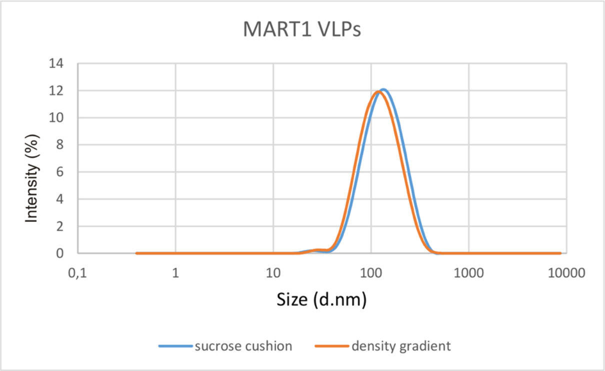

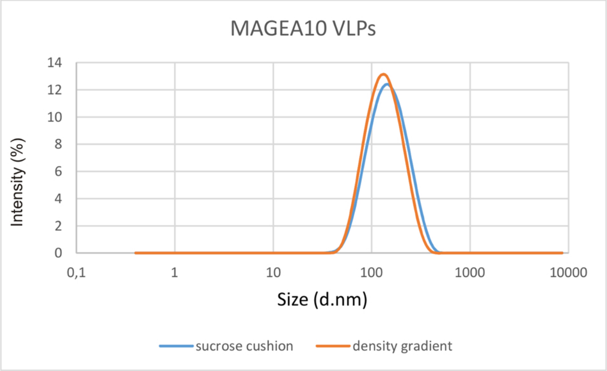

Figure S1. Size distribution of MART1 and MAGEA10 VLPs after purification with ultracentrifugation through 20% sucrose cushion and stepwise density gradient as assessed by DLS. Each curve shows mean of 4 x 10 measurements performed at 22°C.

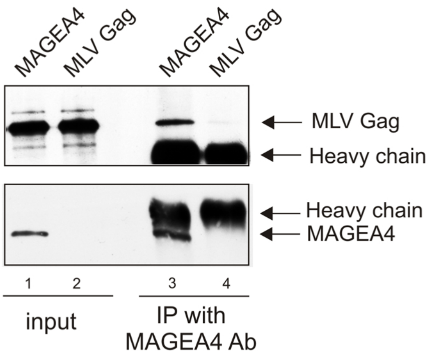

Figure S2. Co-localization of antigene and the MLVgag protein within the VLPs. MAGEA4 VLPs were immunoprecipitated with MAGEA4-specific antibodies and analyzed by immunoblotting with antibodies against MLV Gag and MAGEA4. Input (20%) and pull-down fractions (IP) are shown. 10 µg of VLPs were diluted in TN buffer containing 10% glycerol and 0.5% Tween20 and incubated with 1 µg of MAGEA4-specific antibody for 1 hour at 4°C. Then 5 µl of protein G magnetic beads (Dynabeads) were added, incubated for another hour, washed and analyzed by immunoblotting.
